# St. Louis Clinics

**Published:** 1893-03

**Authors:** Z. F. Lillard

**Affiliations:** Reporter; Tyler, Texas


### ST. LOUIS CLINICS.

Reported for Daniel's Texas Medical Journal, by Dr. Z. F. Lillard, of Tyler, Texas.

ADDISON'S DISEASE.—Martin Meyer; age 55 years, German, single, occupation that of cook. Family history good. Father died at age of 95, mother at age of 84 years. Had three brothers; two are yet living, one died at 55 years of age. No sisters.

Habits—Drinks beer and whisky in moderation; never was drunk. Excessive smoker. Hygienic surroundings unhealthy.

Previous history—Had chills when about 15 years old. No other sickness till 1874, when present trouble began.

Present trouble began with an intense itching, a little over eighteen years ago. Itching extended over entire surface of body, and still persists. Soon after itching began, the skin became of a yellowish hue, and now presents the characteristic bronze color. Up to this time, appetite and general health have been fair. Has had occasional attacks of dizziness. For past nine years patient has suffered from frequent asthmatic attacks. This, with a gradual increasing weariness, is what induced him to come into the hospital. Skin dry; conjunctivæ appear jaundiced. Tongue red, dry, and slightly coated; great thirst; bowels regular. Pulse 80, small and weak; respiration 36; temperature 98.

Physical Examination—Patient's chest presents the characteristic emphysematous appearance. Liver pushed downward, and extends almost to umbilicus. Spleen enlarged. Urine yellowish-brown, and acid; s. g. 1028. No albumen. Biliary reaction.

Diagnosis—Addison's disease, with asthma and emphysema.

The disease of peculiar interest in this case is Addison's. Patient has been in hospital several times in past eighteen years. In 1875, the records show a diagnosis of chronic hepatitis. In 1884, a diagnosis of xanthoma multiplex was made, and this he undoubtedly had. Prof. Hardaway describes some of the peculiarities of patient's skin disease, on page 406 of his Manual of Skin Diseases. At present, patient shows no vestige of this affection.

